# Prediction of drug sensitivity based on multi-omics data using deep learning and similarity network fusion approaches

**DOI:** 10.3389/fbioe.2023.1156372

**Published:** 2023-04-13

**Authors:** Xiao-Ying Liu, Xin-Yue Mei

**Affiliations:** ^1^ Guangdong Polytechnic of Science and Technology, Zhuhai, China; ^2^ Institute of Systems Engineering, Macau University of Science and Technology, Taipa, China

**Keywords:** multi-omics data, drug sensitivity prediction, deep learning, SPCA, similarity network fusion

## Abstract

With the rapid development of multi-omics technologies and accumulation of large-scale bio-datasets, many studies have conducted a more comprehensive understanding of human diseases and drug sensitivity from multiple biomolecules, such as DNA, RNA, proteins and metabolites. Using single omics data is difficult to systematically and comprehensively analyze the complex disease pathology and drug pharmacology. The molecularly targeted therapy-based approaches face some challenges, such as insufficient target gene labeling ability, and no clear targets for non-specific chemotherapeutic drugs. Consequently, the integrated analysis of multi-omics data has become a new direction for scientists to explore the mechanism of disease and drug. However, the available drug sensitivity prediction models based on multi-omics data still have problems such as overfitting, lack of interpretability, difficulties in integrating heterogeneous data, and the prediction accuracy needs to be improved. In this paper, we proposed a novel drug sensitivity prediction (NDSP) model based on deep learning and similarity network fusion approaches, which extracts drug targets using an improved sparse principal component analysis (SPCA) method for each omics data, and construct sample similarity networks based on the sparse feature matrices. Furthermore, the fused similarity networks are put into a deep neural network for training, which greatly reduces the data dimensionality and weakens the risk of overfitting problem. We use three omics of data, RNA sequence, copy number aberration and methylation, and select 35 drugs from Genomics of Drug Sensitivity in Cancer (GDSC) for experiments, including Food and Drug Administration (FDA)-approved targeted drugs, FDA-unapproved targeted drugs and non-specific therapies. Compared with some current deep learning methods, our proposed method can extract highly interpretable biological features to achieve highly accurate sensitivity prediction of targeted and non-specific cancer drugs, which is beneficial for the development of precision oncology beyond targeted therapy.

## 1 Introduction

In the last few years, due to the continuous development of high-throughput bio-data and bioinformatics technologies, people have paid more and more attention to analyze tumor biomarkers and drug targets. The use of genomic data to guide the treatment of cancer patients represents the central principle, which matches patients to specific tumor types and treatments based on the molecularly targeted drugs (Zhang and Yue, 2015) (Kumar-Sinha and Chinnaiyan, 2018) (Chen et al., 2019). Researchers have identified many molecular lesions as triggers that drive cancer, and suggested that each cancer has its own genetic imprint and tumor marker. The corresponding therapeutic drug is designed for a well-studied target that promotes tumor growth (the target can be a protein molecule on the surface or inside the tumor cell, or a gene fragment). However, the drug response and sensitivity to cancer treatment (chemotherapy or targeted drugs) is a complex pharmacology that usually depends on many factors, especially the patient’s genomic profile (Lee et al., 2018). In clinical practice, molecularly targeted drugs are recommended for patients only if the target gene is mutated. However, according to available studies, only about 9% of patients can be identified by known target genes in precision therapy (Min et al., 2018). Additionally, only about 11% of patients can enter clinical trials. Most importantly, only 5% of patients achieve optimal treatment outcomes in precision oncology (Cheng et al., 2018) (Marquart et al., 2018) (Zehir et al., 2017). In consequence, there are limitations in selecting drugs for molecularly targeted therapies based on the genomic status of the patient. Large-scale pharmaco-genomes based on cell lines or patient-derived xenografts (PDX) models in recent years have been working to uncover relationships between multi-omics biosignatures and drugs, aiming to obtain drugs that match tumors. The results of PDX and existing large-scale pharmacogenetic screens of cell lines show that nearly all cancer patients are sensitive to one or more targeted drugs or non-specific chemotherapeutic drugs. As a result, how to accurately match cancer patients with their sensitive drugs is currently a critical research challenge.

According to the previous summary, there are usually two computational and analytical approaches for predicting drug response. The first one is using regression approaches to predict the value of the evaluation criteria of cell lines to drug response, and the second one is classifying the sensitivity of each drug on the basis of cell lines (Ahmadi Moughari and Eslahchi, 2021). Choi et al. presented a computational model based on the elastic network regressions and deep neural networks (Choi et al., 2020). They predicted the probability of drug sensitivity of a specific cell line to a drug based on the similarity of the drug to a reference group. Wang et al. proposed a matrix factorization with similarity regularization model (SRMF) to predict drug response values, which is based on the gene expression similarity of cell lines and pharmacochemical similarity (Wang et al., 2017). In addition, there are many other regression computational methods. When recommending appropriate and effective therapies for cancer patients, it is important to determine the drugs to which they are sensitive. However, even knowing the drug response value itself may not provide additional information in clinical treatment. Therefore, classifying cell lines as sensitive or resistant to each drug is a more straightforward and effective method than regressing their response values. Furthermore, the regression problem could have been transformed into a classification problem by setting a threshold value.

Most studies have shown that gene expression data is the most powerful data type for classifying and predicting drug response (Ding et al., 2016) (Iorio et al., 2016) (Graim et al., 2018) (Koras et al., 2020). In 2014, there were scholars who used baseline gene expression levels and *in vitro* drug sensitivity of cell lines to predict clinical drug response (Geeleher et al., 2014). MJ et al. used gene expression microarrays to assess the prognosis of patients with primary breast cancer (Van De Vijver et al., 2002).

Non-etheless, with the development of next-generation sequencing and mass spectrometry technologies, which accelerates the development of omics research toward quantification and high throughput, there is an increasing need for the ability to fuse biological features to study whole treatment processes. Proteomic, transcriptomic, methylomic, histone post-translational modifications, and microbiomic features all influence the host response to various diseases and cancers. The integration of multi-omics approaches has led to a deeper understanding of disease etiology, where data from a single genomics cannot capture the complexity of all factors associated with understanding a phenomenon (e.g., disease) (Zitnik et al., 2019). Models that integrate multi-omics data to identify patients’ drug sensitivity in advance have become the central object of cancer research (Olivier et al., 2019) (Chaudhary et al., 2018).

Researchers have already proposed some multi-omics machine learning and deep learning methods for drug sensitivity prediction. However, the biomolecular data are often high-dimensional, e.g., methylation data may be 400,000 to 500,000 dimensions while the sample size is only about 1,000. These methods may suffer from overfitting problems and have difficulties in fusing multi-omics data. In addition, the interpretability of deep neural networks is relatively low, and biomedical methods lacking interpretability make it difficult for the reliable diagnoses of doctors. Moreover, the accuracy of these existing models also has some room for improvement.

In response to these challenges, we proposed a novel multi-omics drug sensitivity prediction model (NDSP) based on deep learning and similarity network fusion approaches. The model extracts biomarkers using an improved sparse principal component analysis (SPCA) method for each omics data, and constructs sample similarity networks based on the sparse biomarker matrices, which greatly reduces the dimensionality of multi-mics data and weakens the risk of overfitting in the training process of deep learning. Finally, the fused similarity networks are put into a deep neural network for training and the model can make full use of the high integrability and interpretability of the similarity networks. Compared with some current deep learning methods, our proposed model has the ability to handle high dimensional data and highly interpretable feature selection capabilities. More importantly, the model has higher prediction accuracy than existing models for both targeted and non-specific therapeutics drugs, which is beneficial for the development of precision oncology.

## 2 Related work

### 2.1 Single gene expression data models

A number of researchers have proposed cancer drug sensitivity prediction models based on single genomics data. For example, Ali oskooei et al. proposed a network-based tree integration (netBite) machine learning approach to identify the biomarkers of drug sensitivity using gene expression data. The authors applied the netBite model to a set of GDSC data for 50 anticancer drugs, where Linifanib was able to achieve an accuracy of about 0.7, and demonstrated that netBite outperformed Random Forest in predicting IC50 drug sensitivity, but only for drugs targeting membrane receptor pathways (MRps): iGfR, RtK and eGfR signaling pathways (Oskooei et al., 2019). Gilleher et al. integrated several computational and statistical tools such as linear ridge regression, logistic ridge regression, elastic network and lasso regression to analyze the data of 138 drugs from nearly 700 cell lines to predict drug sensitivity *in vivo*. The experiments proved that ridge regression models trained on GDSC gene expression data could be translated to clinical trial data of Erlotinib, Docetaxel, Bortezomib, and Cisplatin. The paper also indicated that the inclusion of non-breast cancer samples in model training process improves the predictive accuracy of the final model compared with the models trained on breast cancer cell lines only (Geeleher et al., 2014). This gene expression delivery pathway based on ridge regression also roughly predicted the drug response of The Cancer Genome Atlas (TCGA) (Geeleher et al., 2017) (Weinstein et al., 2013).

### 2.2 Multi-omics data models

Due to the large biological system, the single genomics data cannot capture all complex factors related to understanding a biological phenomenon (e.g., disease) (Zitnik et al., 2019). Learning methods that integrate multi-omics data are beginning to be widely used in biology and medicine, such as identification of driver genes (Dimitrakopoulos et al., 2018) (Mo et al., 2013), patient stratification (S Khakabimamaghani et al., 2019), cancer subtype discovery (Liang et al., 2014), patients survival prediction (Chaudhary et al., 2018), and drug sensitivity prediction. More and more multi-omics drug-sensitive datasets are made publicly available, especially in pan-cancer models (Iorio et al., 2016). The application of multi-omics data allows machine learning models to better characterize biological processes from different perspectives (Wang et al., 2014) (Argelaguet et al., 2018).

Ding et al. proposed a data-driven precision medicine approach to learn new biological features from omics data to address the dimensionality challenge. The copy number variation, mutation, and gene expression data were concatenated. The variance-based mixed-fit feature selection was performed using the original omics features as the input to the elastic network approach to predict the binarized IC50 values (Ding et al., 2018). Chiu et al. also proposed an autoencoder-based integrated genomic profiling deep learning model for drug response prediction (Chiu et al., 2019). The model contained three deep neural networks. The first layer was a mutation encoder pre-trained using a large pan-cancer dataset (The Cancer Genome Atlas; TCGA) to abstract the core representation of high-dimensional mutation data. The second layer was a trained expression encoder, and the third layer was a drug response prediction network that integrated the first two sub-networks. Hossein et al. presented a multi-omics drug response prediction model named MOLI based on deep neural network (Sharifi-Noghabi et al., 2019), which integrated three omics data of somatic mutations, copy number aberrations and gene expression data for multi-omics analysis. To address the key challenge of how to integrate diverse data types, the model proposed the first end-to-end post-integration approach. This approach used each histological data type to make separate type-specific neural networks, and every encoding sub-network learned features on behalf of its omics data type. Moreover, the extracted features were connected into the feature representation, which was optimized by a joint cost function made up of a binary cross-entropy loss and a triplet loss, while updating all the data for three omics.

### 2.3 Patient similarity network

Although machine learning methods can handle large-scale data, they are usually considered as black boxes that do not explain well the favorability of specific features for prediction. Interpretability is particularly needed in clinical treatment. Patient similarity networks, a framework that excels in integrating heterogeneous data, handling sparse data, and generating interpretable models, has been applied to several biological fields with good results (Li et al., 2015) (Wang et al., 2014). Pai et al. proposed the interpretable patient classification model (netDx), which was a supervised machine learning approach similar to a recommender system using integrated patient similarity networks (Pai et al., 2019). Patients in unknown states can be grouped according to their similarity to determine its risk of the certain disease. The model integrates six types of data across four cancer types, and the experiment results show that netDx performs significantly better than most other machine learning methods on most cancer types. Compared with traditional machine learning-based patient classifiers, the results of netDx are more interpretable and allow visualization of decision boundaries in the context of patient similarity space.

### 2.4 Limitations of the existing models

The existing deep learning approaches based on multi-omics data still have four major challenges. First, learning new information features from omics data is a key step for model-based drug sensitivity prediction. However, biomolecular datasets tend to be high-dimensional, i.e., with a large number of features and a small number of samples. There is a significant risk of overfitting using deep learning models. Second, deep learning models are a black box, and researchers need to spend a lot of effort to explain what role specific features play in prediction. The black-box approaches are difficult to succeed in the clinical setting because physicians must have an understanding of the underlying relevant features of the disease in order to make a confident and reliable diagnosis. Third, how to integrate different data types is a key challenge in multi-omics analysis, and the main ways are early integration and late integration. In the previously mentioned models that fuse the feature representations learned from each omics before classification, a large number of unaligned gene points are inevitably discarded actively to facilitate feature fusion, leading to data loss problems. Fourth, the results of existing multi-omics drug response prediction methods are unsatisfactory, and there is space for improvement.

## 3 Materials and methods

### 3.1 Datasets

In this study, we utilize the available oncology therapeutic genomic data from the Genomics of Drug Sensitivity in Cancer (GDSC) database. This dataset is widely analyzed by statistical and machine learning approaches for drug sensitivity prediction. For example, cell line similarity and drug similarity based models (Sheng et al., 2015), quantitative structure-activity relationship (QSAR) analysis using kernelized Bayesian matrix decomposition (Ammad-Ud-Din et al., 2014), lasso and elastic network models for predicting drug sensitivity and target identification (Barretina et al., 2012) (Park et al., 2015).

**FIGURE 1:**
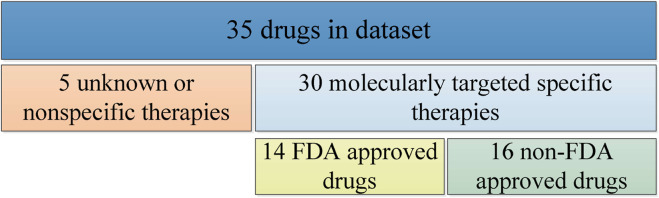
Descriptive classification of drugs in experiments.

We select mutation data, cell line annotation and drug IC50 data from GDSC, including targets, signaling pathways, point mutation and copy number variation information and IC50 values of some genes, and several phenotypes for 518 oncology drugs in 988 cell lines. For drug sensitivity study, we select 35 drugs from the GDSC database as experimental subjects, including 14 FDA-approved targeted therapeutics, 16 drugs with clear targets but not yet approved by FDA, and 5 non-specific cancer therapeutics without targets, as shown in Figure 1.

#### 3.1.1 RNA-sequence data

The RNA-Sequence data is downloaded from the European Bioinformatics Institute (EMBL-EBI): https://www.ebi.ac.uk/arrayexpress/experiments/E-MTAB-3610/. The genomic signature of each cell line contains RNA-Sequence values for 44,421 probes, which is known as whole transcriptome shotgun sequencing (WTSS). It contains transcriptional analysis of 1,000 human cancer cell lines to explore questions such as the state of genomic signature on drug response and whether genomic alterations synergistically explain more of the variation in drug response. RNA Sequencing has been considered an effective method for gene discovery, helping to view different transcripts of genes, post-transcriptional modifications, gene fusions, mutations/SNPs, changes in gene expression over time, and differences in gene expression in different groups.

#### 3.1.2 Copy number aberration (CNA) data

The CNA data is downloaded from Cell Model Passports: https://cellmodelpassports.sanger.ac.uk/downloads. Copy number aberration exists in DNA fragments of natural populations and is a common form of structural genomic variation. Abnormal DNA copy number variation is an important molecular mechanism for many human diseases such as cancer and hereditary diseases. Deletion fragments may contain oncogenes for tumors, while amplified fragments may harbor oncogenes. The genomic signature of each cell line in the collated data contains somatic copy number variation for 21,878 gene loci.

#### 3.1.3 Methylation data

The methylation data is downloaded from Gene Expression Omnibus (GEO): https://www.ncbi.nlm.nih.gov/geo/query/acc.cgi?acc=GSE68379. It reports how cancer-driven alterations detected in 11,215 tumors and 29 different tissues (integrating multiple omics) correlate with responses to 265 compounds in 1,001 cancer cell lines. Cell lines are very similar to tumors in these areas of alteration, and there are many examples of altered genes and pathways conferring drug sensitivity and resistance. Methylation is an important modification of proteins and nucleic acids that regulates the expression and shutdown of genes and is closely associated with many diseases such as cancer, aging, and Alzheimer’s disease, and is one of the key studies in epigenetics. Here we use DNA methylation, which turns off the activity of certain genes, and altered DNA methylation status is prevalent in tumors. The genomic signature of each cell line in our experiments contains the methylation status values of 365,860 CpG loci.

### 3.2 SPCA with feature importance

Hui Zou et al. first proposed the concept of sparse principal component analysis (SPCA) in 2006 (Zou et al., 2006). Suppose ${X\in R}^{m\times n}$ is a data matrix with m features and n samples. The SPCA *via* $L_{0}$ -penalty can be adopted to analyze the matrix:

$$\underset{{\left\|u\right\|}_{2}\leq 1}{maximize}u^{T}XX^{T}u,s.t.\;{\left\|u\right\|}_{0}\leq s,$$ (1)

where *u* is a m×1 vector to represent the first principal component (PC) loading and *s* represents the number of genes retained by the model, ${\left\|u\right\|}_{2}$ represent $L_{2}$ norms (Euclidean norm) and ${\left\|u\right\|}_{0}$ denotes the $L_{0}$ norm, which is equal to the number of non-zero elements of u . Researchers usually use the singular value decomposition framework (SVD) to solve this problem (Lin et al., 2016). Therefore, Formula (1) can also be written as:

$$\underset{{\left\|u\right\|}_{2}\leq 1,{\left\|v\right\|}_{2}\leq 1}{maximize}u^{T}Xv,s.t.{∥u∥}_{0}\leq s,$$ (2)

where v is n×1 vector to represent the first principal component.

The following alternate iterative projection strategy (Journée et al., 2010) is used to solve the problem in Formula (2) until convergence:

$$\begin{array}{c} u=\frac{\hat{u}}{\left\|\hat{u}\right\|},where\hat{u}=P\left(z,s\right),and\;z=Xv\\ v=\frac{\hat{v}}{\left\|\hat{v}\right\|},where\;\hat{v}=X^{T}u \end{array}$$ (3)

where $P\left(z,s\right)$ is called *s*-sparse projection operator. It is a p -dimensional column vector and its i -th ( i=1,2,…,p ) element is defined as follows:

$${\left[P\left(z,s\right)\right]}_{i}=\left\{\begin{array}{cc} z_{i}, & if\;i\in supp\left(z,s\right),\\ 0, & otherwise, \end{array}\right.$$ (4)

where $supp\left(z,s\right)$ denotes the set of indexes of the largest *s* absolute elements of z .

**FIGURE 2:**
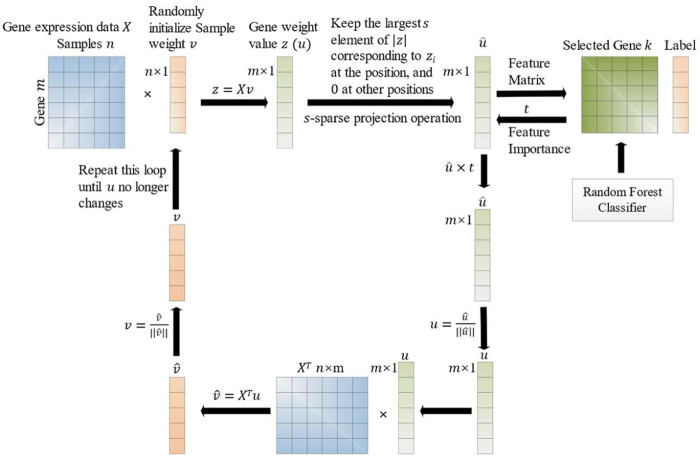
Workflow of SPCA with feature (for example, gene) importance.

Our proposed model uses SPCA for dimensionality reduction and feature selection. SPCA is an unsupervised model, and a feature importance parameter t is calculated based on a classical machine learning model—Random Forest (RF). The unsupervised SPCA method and the supervised classification RF model are combined to evaluate whether the genes in the selected PCs can better predict the sensitivity of the drugs. The workflow of the SPCA with the parameter t is shown in Figure 2.

Suppose there are M features $X_{1},X_{2},...,X_{M}$ , K categories, and D decision trees in the random forest. If the node where the feature $X_{j}$ appears in decision tree, the Gini index score $GI_{j}$ for the feature $X_{j}$ is expressed as follows:

$$GI_{j}=1-\sum_{k=1}^{\left|K\right|}p_{jk}^{2},$$ (5)

where $p_{jk}$ denotes the proportion of the category k for the feature $X_{j}$ .

Suppose $node\left(X_{j}\right)$ is the set that the feature $X_{j}$ appears in the nodes, the importance $t_{jd}$ of the feature $X_{j}$ in the decision tree d :

$$t_{jd}=\underset{node\left(X_{j}\right)\in d}{\sum }GI_{j}.$$ (6)

The importance $t_{j}$ of the feature $X_{j}$ in the random forest:

$$t_{j}=\sum_{d=1}^{D}t_{jd}.$$ (7)

Finally, all the obtained importance scores are normalized to calculate the feature importance:

$$t_{j}=\frac{t_{j}}{\sum_{m=1}^{M}t_{m}},$$ (8)

where M denotes the number of features.

The improvement process of the SPCA with feature importance is described as below. Firstly, the SPCA analyzes the data matrix X to get the *M* largest elements of the absolute value of z , and to make all other positions 0 for spare principal component operation. At this point, the features in the selected principal components are put into the RF classifier for evaluation. The obtained feature importance t updates the data matrix X . This loop is repeated until convergence.

### 3.3 Similarity network fusion

After completing the SPCA with feature importance, we obtain the independent feature matrix for each omics data, the RNA-Sequence matrix ${S\in R}^{a\times n}$ , methylation feature matrix ${M\in R}^{b\times n}$ , CNA feature matrix ${C\in R}^{c\times n}$ , and a,b,c denote the numbers of features retained in each of the three omics. Next, a sample similarity network needs to be constructed for each omics data.

Two main similarity calculation algorithms are used. The Pearson correlation coefficient is suitable for linear continuous variables and the Kendall correlation coefficient is suitable for discrete variables.

For the RNA-Sequence and Methylation data, we use the Pearson correlation coefficient:

$$r_{xy}=\frac{\sum_{i=1}^{a}\left(x_{i}-\bar{x}\right)\left(y_{i}-\bar{y}\right)}{\sqrt{\sum_{i=1}^{a}{\left(x_{i}-\bar{x}\right)}^{2}\sum_{i=1}^{a}{\left(y_{i}-\bar{y}\right)}^{2}}},$$ (9)

where n indicates the number of the samples, $x_{i},y_{i}$ denotes the expression information of the i -th gene locus of sample x,y , $\bar{x},\bar{y}$ denotes the mean gene expression value of sample x,y .

The CNA data are integer discrete representing the variance multiples, so we use the Kendall rank correlation coefficient:

$$\tau =\frac{C-E}{(\begin{array}{c} n\\ 2 \end{array})},$$ (10)

where C denotes the number of pairs of elements in x,y that have consistency; E denotes the number of pairs of elements in x,y that have inconsistency. $(\begin{array}{c} n\\ 2 \end{array})=\frac{1}{2}n\left(n-1\right)$ is the binomial coefficient of the number of ways to select two items.

**FIGURE 3:**
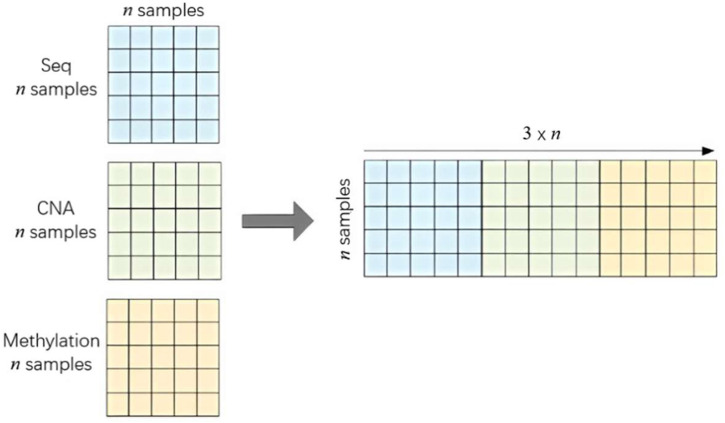
Integration of three similarity networks.

After the similarity calculation, three independent sample similarity matrices are obtained, $S^{\prime }{\in R}^{n\times n}$ , ${M^{\prime }\in R}^{n\times n}$ , ${C^{\prime }\in R}^{n\times n}$ . The data of each omics are turned into n×n size matrices, so that hundreds of thousands of dimensions of omics data reduce to thousands of dimensions of sample similarity matrix, which not only solves the problem of high dimensionality, but also makes the integration operation of multi-omics heterogeneous data much easier. We directly stitch the matrices of several omics data horizontally, as shown in Figure 3, and then use the deep learning model to perform classification operations, instead of turning multi-omics data into one matrix by superposition. This can avoid the information loss during fusion of the multiple omics data.

### 3.4 Deep learning approach

**FIGURE 4:**
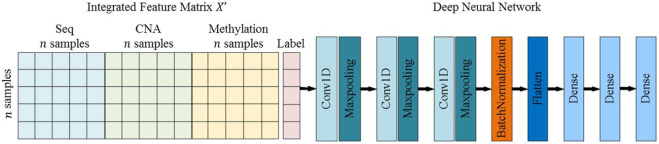
Framework of deep learning approach.

We construct a simple 7-layer deep neural network model and put the n×3n fused similar networks into it for training (Figure 4).

This neural network contains three one-dimensional convolutional layers, each convolutional layer is followed by a max pooling layer, and a batch normalization layer added after the last convolutional layer. In addition, the first two fully connected layers use “relu” as the activation function while the third fully connected layer uses “softmax”. The cross-entropy loss function is used, which is a commonly measurement in dealing with classification problems.

## 4 Experiment results

The deep learning autoencoder model (Chiu et al., 2019), in which the mutation encoder and gene expression encoder were linked the prediction network. The multi-omics post-integration with deep neural networks model (MOLI) (Sharifi-Noghabi et al., 2019) takes somatic mutation, copy number aberration and gene expression data as input, and integrates them for drug response prediction. We conduct experiments on these two deep learning models and the interpretable patient classification model using an integrated patient similarity network (netDx-RF, netDx-EN, netDx-AdaBoost, netDx-SVR, netDx-KNN) (Pai et al., 2019) to compare the results with our proposed model NDSP.

The 35 drugs we selected include 30 targeted drugs and 5 non-targeted chemotherapy drugs, with the targeted drugs divided into 14 FDA-approved drugs in clinical use and 16 FDA-unapproved drugs. The results are evaluated using the sensitivity, specificity, precision, accuracy and F1-score of the model as indicators. Finally, we use the metascape platform to perform enrichment analysis of targets retained by our proposed method NDSP during feature selection and analyze the association and biological significance of these targets with that drug and disease.

In the data preprocessing step, we collected and classified the multi-omics samples (cell lines) into sensitive and non-sensitive classes based on the binarized IC50 values of each specific drug. The unsupervised SPCA in our proposed model NDSP is first used for dimensionality reduction and feature selection. At this time, the PCs based on the SPCA may not relate with the specific drug. Therefore, the supervised model Random Forest (RF) is combined to evaluate whether the genes in the selected PCs could better predict the sensitivity of the specific drug. The feature importance parameter t is calculated based on the classification results of the RF. By updating the feature importance *t* and repeating the loops of SPCA and RF, the genes in the selected PCs can strongly correlate with the sensitivity of the specific drug.

### 4.1 Results of targeted therapy drugs

The mean values of each metric for our proposed method NDSP and the seven baseline models in the 30 targeted drug trials are shown in Table 1.

**TABLE 1:** Index mean of 8 models on targeted therapy drugs.

	Sensitivity	Specificity	p-0	p-1	Precision	Accuracy	F1score-0	F1score-1	F1score -macro avg
NDSP	**0.91**	0.91	**0.91**	**0.89**	**0.90**	**0.90**	**0.90**	**0.90**	**0.90**
MOLI	0.76	0.86	0.82	0.80	0.82	0.82	0.78	0.83	0.80
Autoencoder	0.42	0.59	0.46	0.49	0.48	0.64	0.39	0.53	0.46
netDx-RF	0.68	**0.92**	0.87	0.80	0.84	0.83	0.74	0.85	0.80
netDx-EN	0.53	0.67	0.64	0.68	0.66	0.69	0.54	0.63	0.58
netDx-AdaBoost	0.62	0.86	0.79	0.74	0.76	0.77	0.69	0.79	0.74
netDx-SVR	0.58	0.71	0.75	0.73	0.74	0.72	0.60	0.68	0.64
netDx-KNN	0.77	0.49	0.59	0.73	0.66	0.67	0.67	0.57	0.62

(p-0 denotes precision of classifying class 0; p-1 denotes the precision of classifying class 1; F1score-0, denotes the F1-score of classifying class 0; F1score-1, denotes the F1-score of classifying class 1). The bold values mean the best results.

As can be seen from Table 1, the average sensitivity and specificity of NDSP can reach 91% and 91%, respectively, and basically exceed the baseline models in each index. Although the specificity is a little lower than the netDx model using the RF classifier, but the sensitivity is 23% higher than the netDx model. Overall, the best performance among the seven baseline models is still the MOLI model, but its average sensitivity, specificity, precision, accuracy and F1 scores can only reach 0.76, 0.86, 0.82, 0.82, and 0.8, respectively.

**FIGURE 5:**
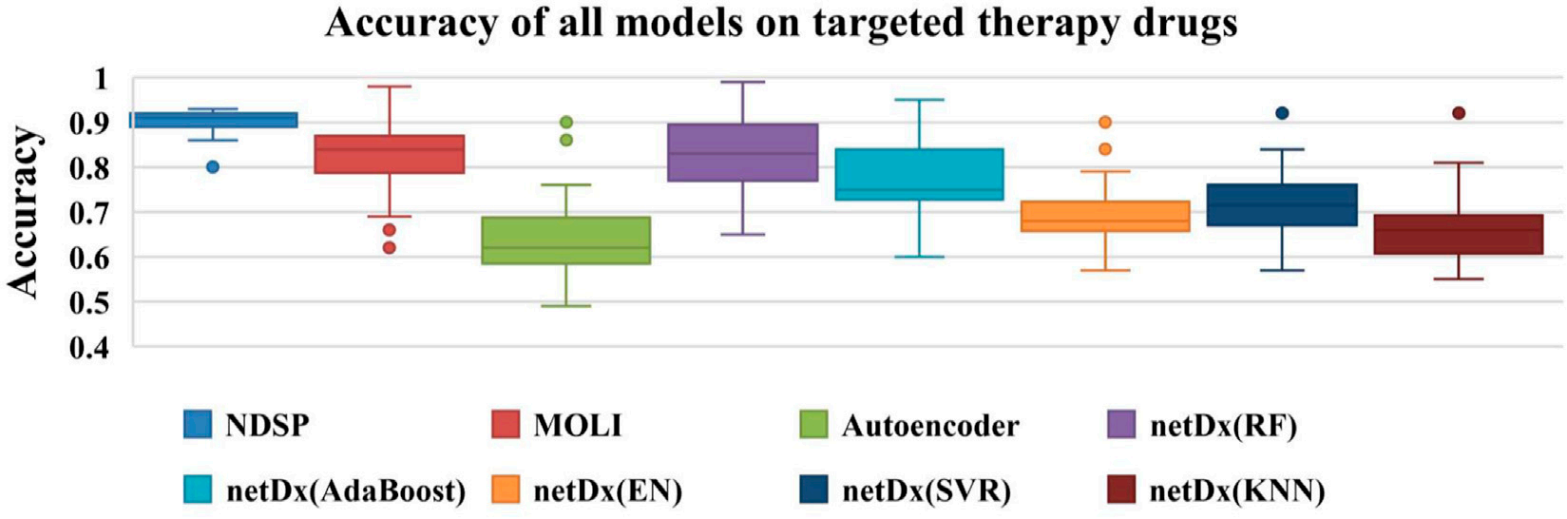
Accuracy of all 8 models on 30 targeted therapy drugs (the points outside the boxplot are outliers).

As shown in Figure 5, the accuracy of NDSP basically reaches 0.9. The netDx model tests five classifiers: EN, SVR, KNN, AdaBoost, and RF. We can see that the accuracy of netDx with RF classifier is the best, but it still has some distance from our proposed model NDSP. NDSP has the highest overall accuracy and fewer outlier points, indicating stable performance. In general, the experiment results of NDSP are the best in regards to the accuracy.

**FIGURE 6:**
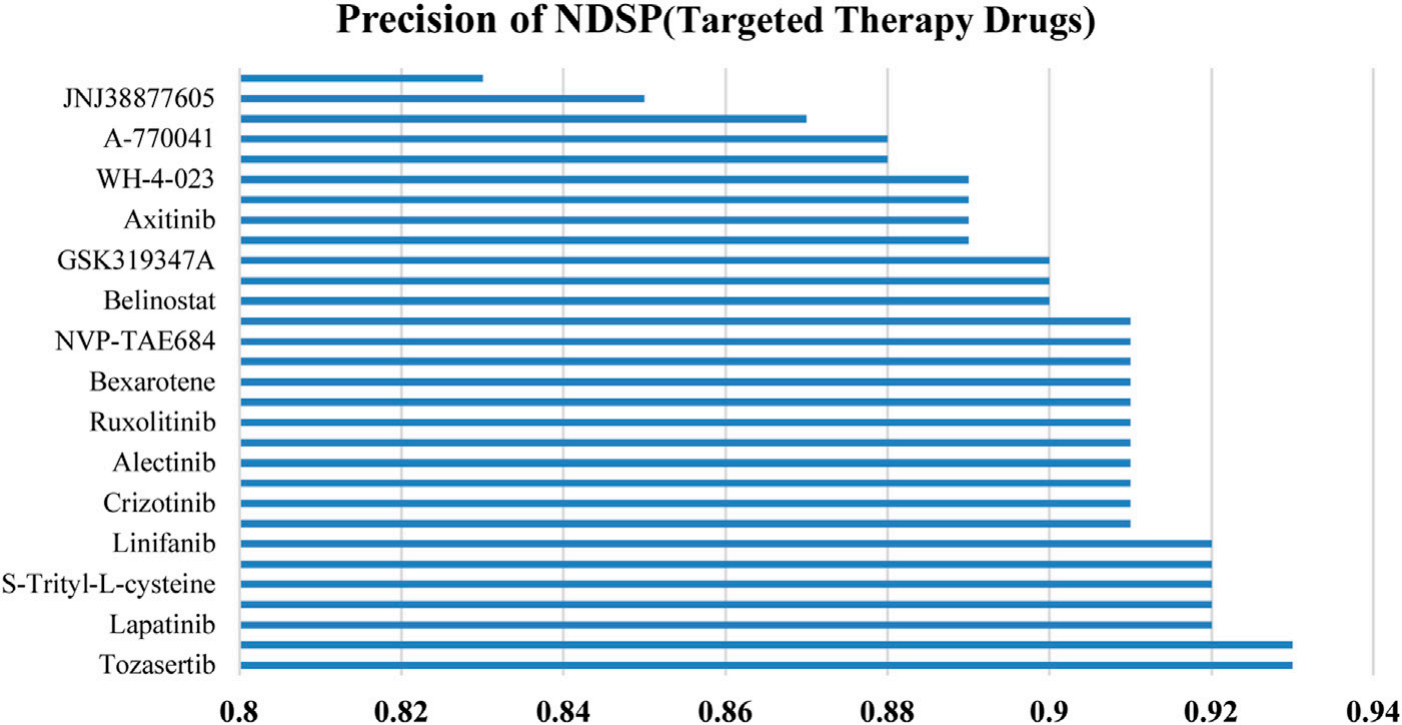
Precision of NDSP on targeted therapy drugs.

Figure 6 shows the prediction precision of NDSP trained on 30 targeted therapy drugs. It can be seen that the precision of our model on each targeted drug is above 0.82 and is mainly concentrated on 0.88 to 0.93.

### 4.2 Results of non-targeted therapy drugs

To verify whether our proposed model NDSP can work in precision oncology beyond targeted therapy, we conduct experiments on 5 non-specific therapeutic drugs. The mean values of each index for the eight models in the five experiments with non-targeted drugs are shown in Table 2.

**TABLE 2:** Index mean of 8 models on non-targeted therapy drugs.

	Sensitivity	Specificity	p-0	p-1	Precision	Accuracy	F1score-0	F1score-1	F1score -macro avg
**NDSP**	**0.90**	0.92	**0.92**	**0.89**	**0.91**	**0.91**	**0.91**	**0.9**	**0.91**
**MOLI**	0.69	0.80	0.62	0.87	0.75	0.78	0.64	0.83	0.73
**Autoencoder**	0.23	0.85	0.39	0.73	0.56	0.65	0.19	0.75	0.47
**netDx-RF**	0.34	**0.99**	0.88	0.83	0.86	0.84	0.48	0.91	0.69
**netDx-EN**	0.21	0.91	0.58	0.76	0.67	0.75	0.27	0.83	0.55
**netDx-AdaBoost**	0.38	0.91	0.66	0.80	0.73	0.78	0.48	0.85	0.66
**netDx-SVR**	0.30	0.92	0.59	0.78	0.69	0.77	0.37	0.84	0.61
**netDx-KNN**	0.56	0.64	0.38	0.79	0.59	0.64	0.45	0.70	0.58

The bold values mean the best results.

The comparison results in Table 2 are similar to the experiments on targeted drugs. NDSP could achieve an average sensitivity and specificity of 0.9 and 0.92 respectively on non-targeted therapy drugs, and basically exceed the baseline models in all metrics. The specificity of the netDx model using the RF classifier is higher than that of the NDSP model, but the sensitivity is only 0.34, which is 66% lower than that of the NDSP model. In the non-targeted drug experiments, the seven baseline models perform much worse than in the targeted drug experiments, probably because of the low number of experiments. But the NDSP model still maintains good performance. Overall, the best performance among the seven baseline models is still the MOLI model, but its average sensitivity, specificity, precision, accuracy and F1 scores are only 0.69, 0.80, 0.75, 0.78, and 0.73, respectively, which are still some distance from NDSP. The specificity of netDx models using RF, EN, AdaBoost and SVR is generally good, but the sensitivity is poor in all cases. The Autoencoder model also has imbalanced sensitivity and specificity.

**FIGURE 7:**
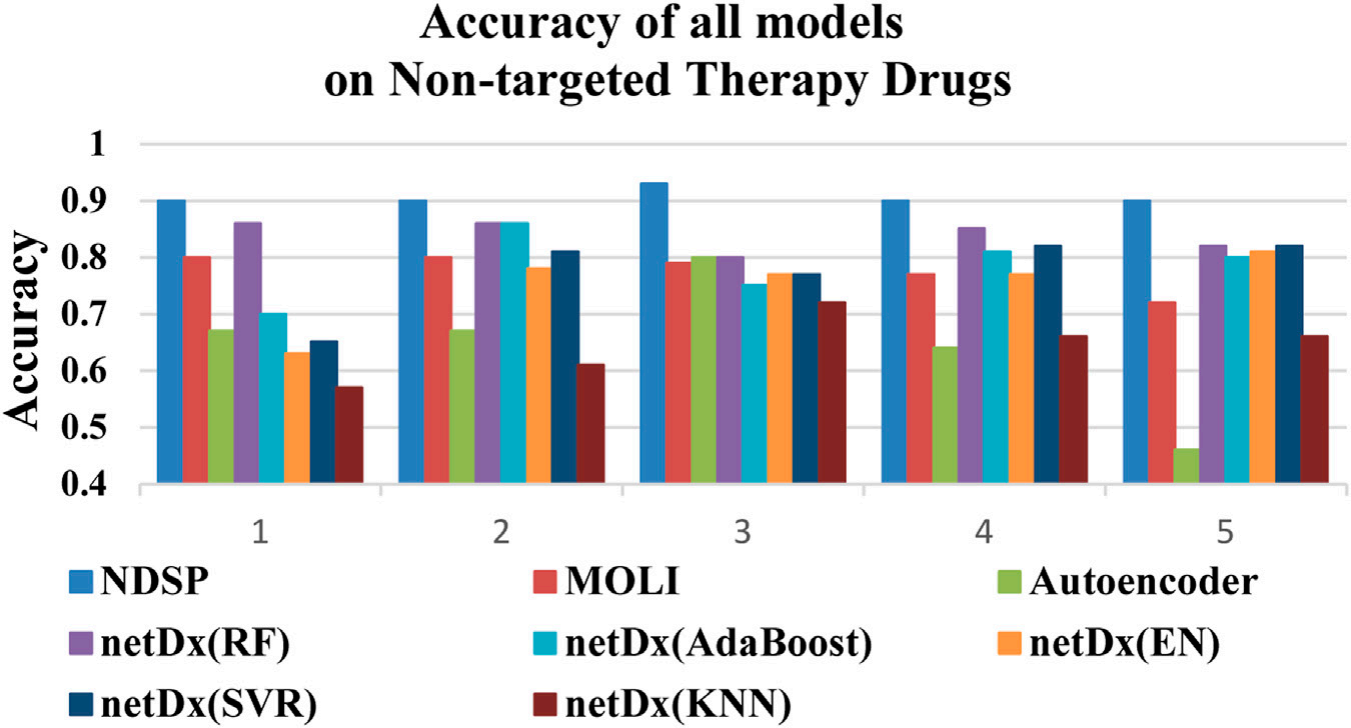
Accuracy of all 8 models on 5 non-targeted therapy drugs (1: FH535, 2: Vinblastine, 3: Z-LLNIe-CHO, 4: Imatinib, 5: Vinorelbine).

Figure 7 shows that the NDSP model has the highest prediction accuracy, reaching above 0.9 with small variation. Among the seven baseline models, the netDx model using RF is the best. But the accuracy is only 0.8 to 0.9, which is not as good as the NDSP model. The other models have accuracy between 0.4 and 0.85 with large variability.

**FIGURE 8:**
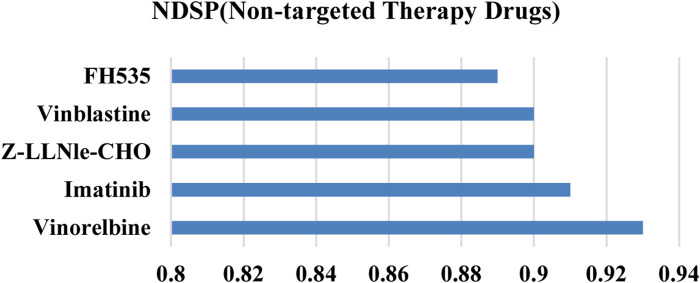
Precision of NDSP on non-targeted therapy drugs.

Figure 8 demonstrates that the prediction precision of our model on all 5 non-targeted drugs is above 0.88. Overall, the results of NDSP are optimal for both molecularly targeted and non-specific drugs, which indicates that NDSP is generalizable and can be useful for precision therapy beyond targeted therapy.

### 4.3 Enrichment analysis

**FIGURE 9:**
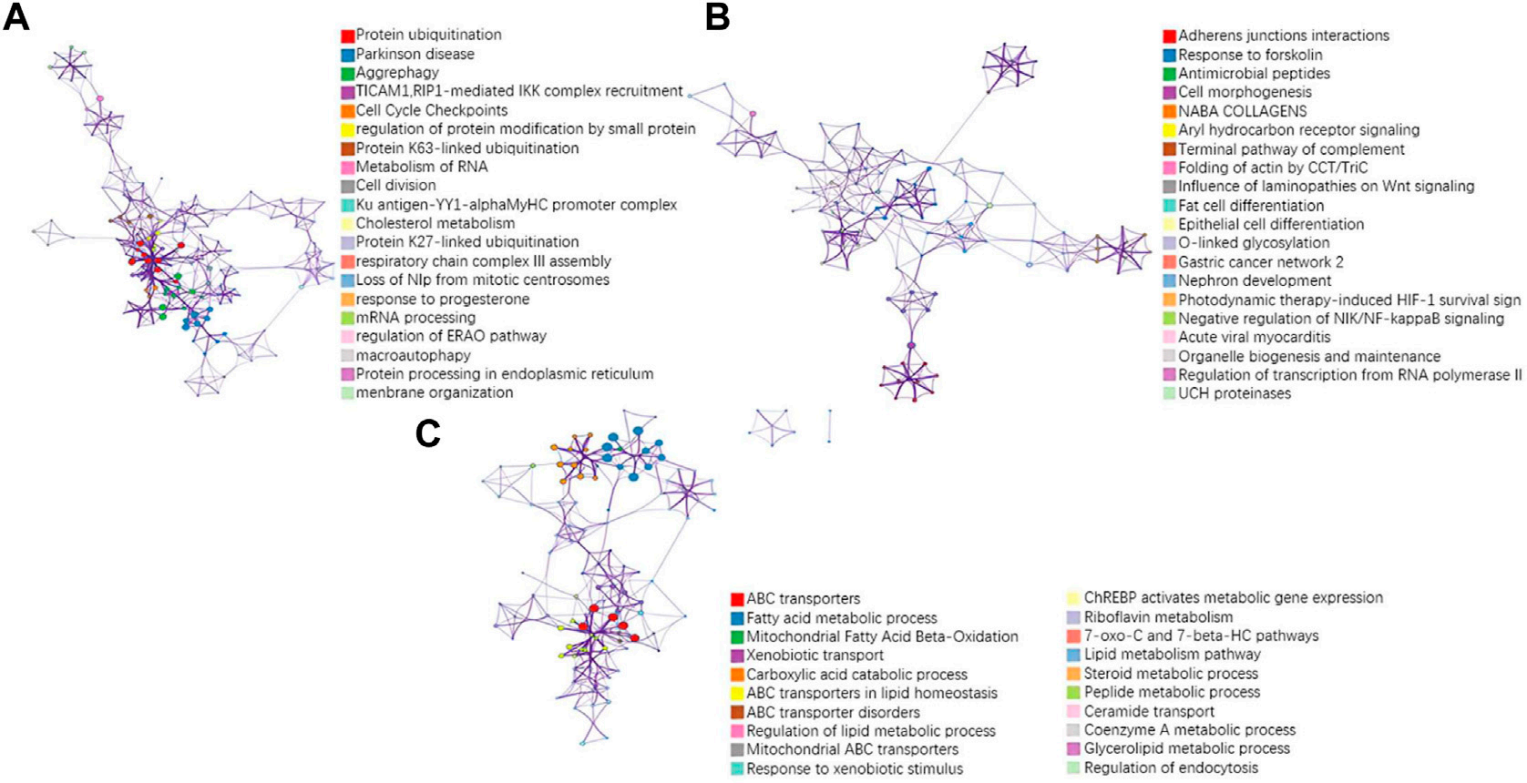
Results of pathway enrichment analysis of the drug Alectinib. **(A)** pathway results for the first PC of seq omics data; **(B)** pathway results for the first PC of CNA omics data; **(C)** pathway results for the first PC of methylation omics data.

To further validate the biological interpretability of our proposed model NDSP, we perform a biological enrichment analysis using the results of the multi-omics gene selection of the new model in Alectinib drug. The first principal component is obtained from the data of each omics in a SPCA module with the addition of a classifier. Drug Alectinib is mainly used for the treatment of non-small cell lung cancer and blocks the activity of ALK. The results of the pathway enrichment analysis are shown in Figure 9.

**FIGURE 10:**
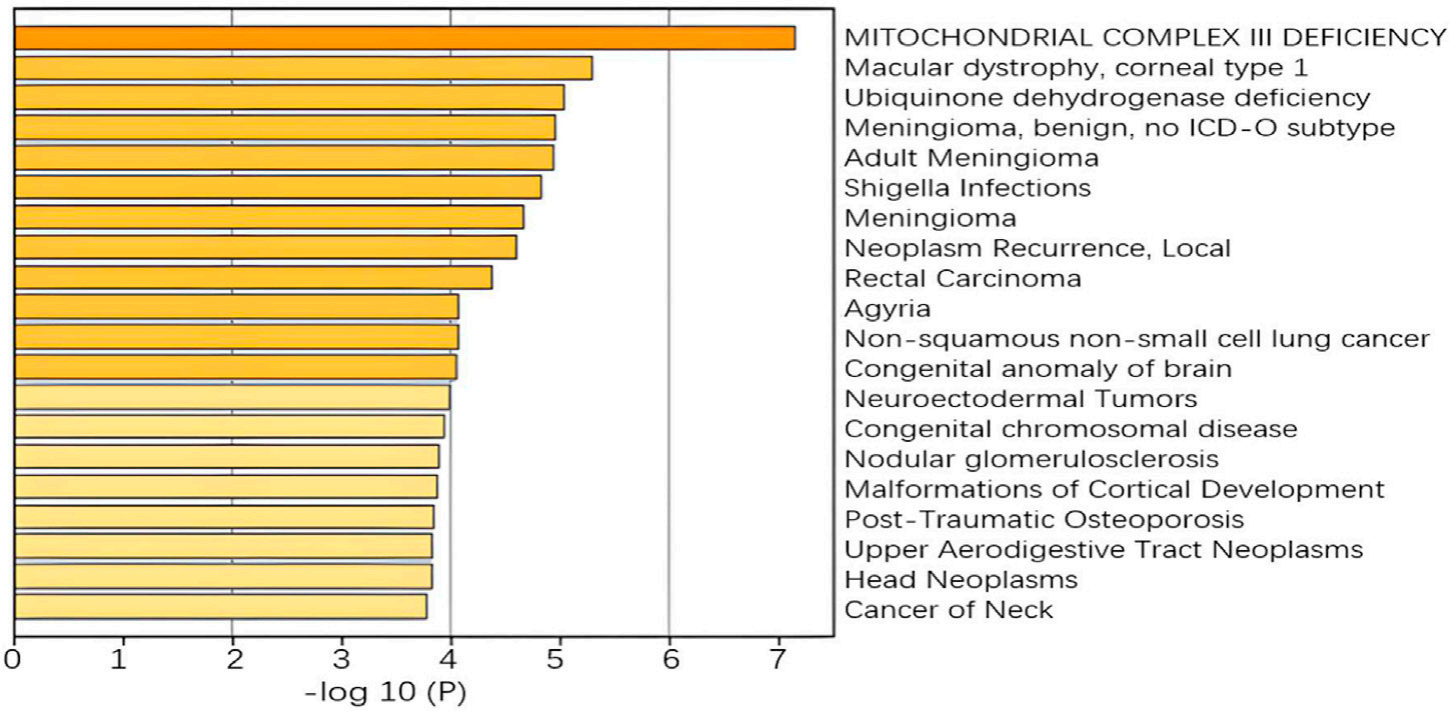
Enrichment analysis in DisGeNET.

A more concentrated distribution of gene sites selected by our model would indicate that the gene set is associated with a specific function or phenotype and is able to select pathways and gene sites that are more relevant to lung cancer. For example, in the RNA-seq omics results, the ERAD pathway corresponding to GO: 1904292 is highly associated with heritable lung disease regulatory mechanisms. And analysis in the integrated platform for integrating information on human disease-associated genes and variants (DisGeNET) shows that the selected gene sites are associated with non-small cell lung cancer, as shown in Figure 10. In CNA omics data, HIF-1 survival signaling corresponding to WP3614 in WikiPathway is associated with tumor development.

## 5 Discussion

We proposed a novel drug sensitivity prediction model (NDSP) that combines biological multi-omics data, SPCA with classical machine learning classifier, patient similarity networks and deep learning. We use data from three omics: RNA sequencing data, Copy Number Aberration data and DNA methylation data. The SPCA with feature importance method is used for feature selection. Then we use patient similarity network to measure the similarity of the three omics feature matrices separately to obtain three matrices of n×n size, which is very efficient at integrating heterogeneous data and can generate interpretable models. This greatly reduces the size of the matrices, making hundreds of thousands of dimensions of omics data into a few thousand dimensions of sample similarity matrices to solve the high dimensionality problem of data. Moreover, it also makes the integration of multi-omics heterogeneous data easier. Finally, the three similarity networks are spliced horizontally and put into a deep neural network model for classification prediction.

We have conducted experiments using both targeted and non-targeted drugs. The available results show that our proposed model NDSP outperforms classical machine learning and deep neural network models in terms of sensitivity, specificity, accuracy, precision and F1-score. More importantly, the drugs selected for the experiments include both targeted and non-specific therapeutic drugs, which implies that the model has a certain degree of generality, and can be useful in precision therapy beyond traditional precision oncology and targeted therapy. The results of the enrichment analysis also show that the targets selected by NDSP are biologically interpretable and have some correlation with the corresponding drugs and diseases. This will guide physicians in selecting optimal treatment options while minimizing the negative effects associated with ineffective treatments, thereby fulfilling the promise of precision therapy.

## Data Availability

The datasets presented in this study can be found in online repositories. The names of the repository/repositories and accession number(s) can be found in the article/supplementary material.
